# Developing Abstract Representations of Passives: Evidence From Bilingual Children’s Interpretation of Passive Constructions

**DOI:** 10.3389/fpsyg.2020.545360

**Published:** 2020-12-11

**Authors:** Elena Nicoladis, Sera Sajeev

**Affiliations:** Department of Psychology, University of Alberta, Edmonton, AB, Canada

**Keywords:** bilingual first language acquisition, cross-linguistic transfer, passive constructions, positive transfer, usage-based theories of language

## Abstract

According to usage-based theories, children initially acquire surface-level constructions and then abstract representations. If so, bilingual children might show lags relative to monolingual children early in acquisition, but not later on, once they rely on abstract representations. We tested this prediction with comprehension of passives in 3- to 6-year-old children: French–English bilinguals and English monolinguals. As predicted, younger bilingual children tended to be less accurate than monolingual children. In contrast, the older bilingual children scored equivalently to monolinguals, despite less exposure to English. When the children made errors, the bilingual children were more likely to interpret the subject as the agent of the action than the monolingual children. These results are consistent with the argument that children develop increasingly abstract representations of linguistic constructions with usage. They further suggest that bilingual children might catch up with monolingual through use of selective attention and/or a semantic bias.

## Introduction

According to usage-based accounts of language acquisition (Tomasello, 2000, 2003; Bybee, 2010), children first learn surface forms of language (i.e., as presented in the input) before generalizing to more abstract forms of representation. Abstract representation allows children to generate novel constructions that are nonetheless grammatical (Tomasello, 2000). The process of abstraction is thought to be both gradual and conservative, as well as highly linked to frequency of usage (Marchman and Bates, 1994; Matthews et al., 2005; Ambridge et al., 2015).

Bilingual children use each of their languages less, on average, than same-aged monolinguals (see Unsworth, 2016). Usage-based accounts would therefore predict that bilinguals should lag in language acquisition relative to monolinguals. Indeed, studies have shown that bilinguals often lag behind monolinguals in at least one language in terms of vocabulary (Oller et al., 2007; Bialystok, 2009; Bialystok et al., 2010; Scheele et al., 2010; Hoff et al., 2012) and morphology (Nicoladis and Marchak, 2011; Nicoladis et al., 2012). Even for monolinguals, frequency plays an important role in the acquisition of vocabulary (Goodman et al., 2008) and morphology (Marchman, 1997), so it is not surprising to see lags among bilinguals in these domains.

Usage-based approaches also predict lags among bilinguals in syntactic acquisition. However, some research shows that young bilingual children do not lag behind monolinguals in syntactic acquisition, including in aspects where the two languages differ structurally, such as in their word order (Paradis and Genesee, 1996; Serratrice et al., 2004; Paradis et al., 2005-2006). However, these studies have often relied on data drawn from children’s spontaneous speech. Usage-based approaches could explain bilingual children’s high degree of accuracy in terms of learning surface-level representations. If so, then the apparent commensurate performance would not truly reflect bilinguals’ syntactic development. Lags in bilinguals’ syntactic acquisition might be observable in experimental tasks, tapping children’s ability to process novel constructions and therefore a more abstract level of re(presentation). Indeed, bilingual children show some delays in syntax relative to monolingual children on experimental tasks, like elicitation (Pérez-Leroux et al., 2009).

Regardless of the theoretical framework adopted, researchers generally agree that bilingual children can eventually rely on language-independent representations of syntax [e.g., Universal Grammar (Gawlitzek-Maiwald and Tracy, 1996; Kupisch, 2006) and constructivist and usage-based accounts (Tomasello, 2000; Gathercole, 2007), the Competition Model (Döpke, 1998)]. Usage-based approaches predict developmental changes in the degree of abstraction of linguistic representation, from surface-level and language-specific to abstract and language-independent. There is evidence among adult language learners for this change (Bernolet et al., 2013). The purpose of the present study was to test for this developmental change in bilingual children.

One way to detect bilingual children’s reliance on abstract shared representation of language is through cross-linguistic influence. Cross-linguistic influence refers to processing language in such a way that shows influence from the other language (Serratrice, 2013). For example, a French–English bilingual child might produce an adjective following a noun in English (like *the hat purple*) because adjectives often follow nouns in French (Nicoladis, 2006). Cross-linguistic influence must reflect an abstract representation of syntax that is at least somewhat shared across languages, such as overlap in word order (Müller and Hulk, 2001). For this reason, usage-based approaches predict that cross-linguistic influence should increase as children age and use the languages more. To date, results have not consistently supported that prediction. Some studies have shown that cross-linguistic influence decreases with increasing proficiency and/or age (Serratrice et al., 2004; Zwanziger et al., 2005; Nicoladis, 2006, 2012; Hacohen and Schaeffer, 2007), while other studies have shown the opposite pattern (Nicoladis and Gavrila, 2015) or that proficiency interacts with other variables to predict cross-linguistic influence (Navés et al., 2005; Kupisch, 2007; Sorace et al., 2009; Mykhaylyk and Ytterstad, 2017). However, these studies have concerned aspects of language in which cross-linguistic influence resulted in errors or infelicitous constructions. As there may be other reasons for children’s errors, the present study focused on a linguistic construction for which cross-linguistic influence would increase children’s accuracy (also known as positive transfer; see Chan et al., 2017).

The goal of the present study was to test the prediction that bilingual children will show more positive transfer with passive constructions in English and French as they get older. Full passives are constructed in the same way in both English and French and can be word-for-word translations. This quality of passive constructions in these two languages could allow for positive transfer once children represent these constructions in an abstract, language-independent way.

### Passive Constructions

In passive constructions, the patient of an action is the subject of the sentence and the agent of the action an optional adjunct. Within languages, there are often several different forms of passives (Creissels, 2001). We focus here on the full passive, with agent and patient supplied, because these constructions are formed identically in both English and French (e.g., *la bouteille était remplie par la fille* translates word for word to *the bottle was filled by the girl*).

The ability to comprehend and produce constructions in the passive voice is difficult for both English-speaking children (Horgan, 1976; Lempert, 1978; Maratsos et al., 1985; Vasilyeva et al., 2006) and children acquiring Romance languages like French (Jakubowicz and Seguí, 1980) and Spanish (Estevan, 1985; Pierce, 1992). In interpreting passive constructions, children often interpret the subject as the agent (Lempert, 1978; Jakubowicz and Seguí, 1980). For example, in the passive construction, *the monkey was seen by Sandra*, young children would interpret *monkey* as the subject and *Sandra* as the object of an active sentence. Their interpretation of the sentence then becomes *the monkey saw Sandra.* Children continue making these errors of transposition through to at least 8 years of age (Horgan, 1976; Estevan, 1985).

Children can start to show evidence of abstract knowledge about passives as young as 3 years of age (Messenger et al., 2011) but continue to get better at abstraction as they get older (Savage et al., 2003; Vasilyeva et al., 2010). One study showed that bilingual children surpassed monolingual children in their ability to interpret passive constructions by the age of 9 years (Filippi et al., 2015). These results are consistent with the argument that older bilingual children can understand passives based on language-independent, abstract representations.

### This Study

The purpose of this study is to test the prediction that bilingual children will show greater positive transfer at an older age (5–6 years of age) than at a younger age (3–4 years of age). This prediction was generated from usage-based accounts of language acquisition. According to these accounts, when children are younger, they represent surface-level (and therefore language-specific) knowledge. Therefore, children’s experience with a particular language should be highly correlated with their accuracy in interpreting passive constructions. Since bilingual children have had less experience, on average, with each language than monolinguals, the younger children should show delays relative to monolinguals. As children get older, they construct abstract, language-independent representations. Older bilingual children should show positive transfer from their other language; in other words, they should interpret passive constructions at least as accurately as monolinguals, despite less exposure.

We also tested the kinds of errors made by the children. The children were asked to interpret passive sentences by picking one of three pictures that corresponded to the meaning of the sentences. One of the distractors was always a picture showing the subject of the passive sentence as the agent of the action. The other distractor depicted the same characters engaged in an action that was not named in the sentence. Given that the bilinguals’ vocabulary size in one language would likely be lower than that of monolinguals’, the monolinguals might be more likely to make transposition errors than bilinguals. That is, since the monolinguals were more likely to be familiar with the words in the sentences, they would be particularly likely to pick a picture corresponding with the subject as the agent. In contrast, because of their lesser familiarity with the words, the bilinguals might pick a picture corresponding to the incorrect activity.

## Materials and Methods

### Participants

All children were between 3 and 6 years of age, living in Canada, recruited through daycares and preschools, and deemed typically developing by their parents and educators. A total of 62 French–English bilingual children participated in this study. Most of the children can be characterized as simultaneous bilinguals, having heard both languages starting at the age of 1 year or younger. There were three children whose age of onset of acquisition of French was between 2 and 3 years and four children whose age of onset of acquisition of English was between 2 and 4 years. The children with later onset were not outliers in any of the measures included in the present study and so were included in all analyses.

A total of 62 age-matched English monolinguals living in Canada also participated in this study. The data were drawn from a database of 79 children. The 62 children were selected as being the closest age match to the bilingual children.

We analyzed the results both with age as a continuous measure and as a categorical variable. To construct the younger and older age groups, we split the groups at the median age of 58 months. Table 1 summarizes the background characteristics of the age and language groups.

**TABLE 1:** Background characteristics of participants.

	**Younger**		**Older**	
	**Bilinguals**	**Monolinguals**	**Bilinguals**	**Monolinguals**
*N*	33	33	29	29
Age range	46–58	47–58	59–82	59–78
Average (SD) age	53.6 (3.4)	54.0 (3.0)	65.1 (6.0)	65.4 (5.0)
#Girls/boys	20/13	17/16	15/14	15/14
PPVT-raw	54.0 (23.1)	78.6 (19.2)	74.3 (17.9)	90.9 (27.3)
PPVT-norm	96.2 (21.2)	116.9 (13.8)	102.7 (12.8)	116.5 (20.2)
EVIP-raw	39.4 (21.7)	n/a	57.6 (22.0)	n/a
EVIP-norm	94.9 (22.6)	n/a	101.3 (22.6)	n/a
Ratio PPVT/EVIP	1.1 (0.4)	n/a	1.1 (0.4)	n/a

*Age is in months. PPVT, Peabody Picture Vocabulary Test; EVIP, Échelle de Vocabulaire en Images Peabody; Ratio PPVT/EVIP, the normed PPVT score divided by the normed EVIP score.*

### Materials and Procedure

For the bilingual children, there were two language sessions: one in English and the other one in French. The monolingual children did the tasks once, in English. The language sessions for the bilinguals were scheduled on different days, usually about a week apart, with different experimenters. The experimenters were native speakers of the target language of the session and spoke entirely in that language during respective sessions. The order of the language sessions was counterbalanced.

In each session, children were given a battery of language and cognitive tasks. The order of the tasks was determined by the experimenter, depending on the child’s level of engagement and willingness to respond. Most often the sessions started off with the more passive tasks, such as receptive vocabulary tests, where children are simply invited to point to a picture corresponding to a word provided by the experimenter. Later in the session, the experimenter would present tasks requiring children’s active production, such as storytelling. We present here the results only for the tasks relevant to the research questions: the receptive vocabulary tests and the test of comprehension of passive constructions.

In the present study, we estimated exposure time to a particular language with vocabulary scores because previous studies have shown strong correlations between vocabulary scores and exposure time to each language in bilingual children (Thordardottir, 2011). All children were invited to take the receptive vocabulary test in English, the Peabody Picture Vocabulary Test III (PPVT; Dunn and Dunn, 1997). In the French session, the children took the French version of this test, the *Échelle de vocabulaire en images Peabody* (EVIP; Dunn et al., 1993). Both the PPVT and EVIP are standardized tests and were administered according to the examiner’s manual. Both tests are standardized so that a normed score of 100 (with a SD of 15) represents age-typical performance. The scores were also calculated according to the manuals. We present both the raw scores and normed scores for both language groups (in Table 1). In order to calculate relative proficiency, we present the ratio of the PPVT normed scores divided by the EVIP normed scores. Thus, a one would represent fairly balanced vocabulary scores. As can be seen in Table 1, on average, the bilingual children were fairly balanced in their vocabulary scores in their two languages, with no difference by age group. In the analyses including vocabulary, we used the raw vocabulary scores (rather than the normed scores) because we were interested in the children’s total vocabulary, not how their vocabulary compared with that of other children of the same age.

The children’s comprehension of passives was measured in English by their performance on passive constructions in Section G (complex sentences) of the Comprehension part of the Reynell Developmental Language Scales III (Edwards et al., 1997). Section G is composed of 10 complex sentences, six of which are in passive voice, such as *The mother is fed by the baby* (see the Appendix for complete list). All of the passive constructions had animate agents and patients so should be challenging for children within this age range (Horgan, 1976). To demonstrate their comprehension of a passive construction, children were presented with three pictures and asked which one corresponded to the sentence. One picture depicted the target (e.g., the baby feeding the mother), another the situation if the agent and the patient were reversed (e.g., the mother feeding the baby), and the third with the agent and patient performing some other action (e.g., the mother hugging the baby). To measure the bilingual children’s comprehension of passives in French, a translated version of the Reynell constructions was presented to them. The exact wording of the passives in French can be found in the Appendix.

### Coding and Analysis

All monolingual children performed all the tasks. Five bilingual children declined our invitation to take the PPVT, two the EVIP, and one the passive task in French. We include these children in the analyses whenever the analyses do not critically involve these measures.

For each child, we calculated the ratio correct in each language out of the total number of items that each of the children answered. For each language, chance was 0.33 (the children had three options, so random choosing should result in one-third correct).

To test for the children’s errors, we calculated the percentage of their errors that were transposition errors (rather than a picture of an irrelevant activity). Not all the children made errors, so we report the exact number of participants included in the analyses below. All statistical analyses were carried out in SPSS.

## Results

The average vocabulary scores for the bilinguals and monolinguals are summarized in Table 1. On a 2 × 2 (Age Group × Language Group) ANOVA on the raw scores of the PPVT, the younger children scored 16.34 lower than the older children, *F*(1,115) = 15.85, *p* < 0.001, η^2^_*p*_ = 0.121 (95%CI of this difference [8.17, 24.36]). The bilinguals scored significantly lower (by 21.09) than monolinguals on the English vocabulary test, *F*(1,115) = 25.40, *p* < 0.001, η^2^_*p*_ = 0.181 (95%CI of this difference [12.50, 28.69]), but there was no interaction between language group and age group, *F*(1,115) = 0.45, *p* = 0.33, η^2^_*p*_ = 0.008. The main effect for Language Group supports our assumption that the bilinguals had less exposure to English than the monolinguals.

### Passives: Accuracy

**FIGURE 1:**
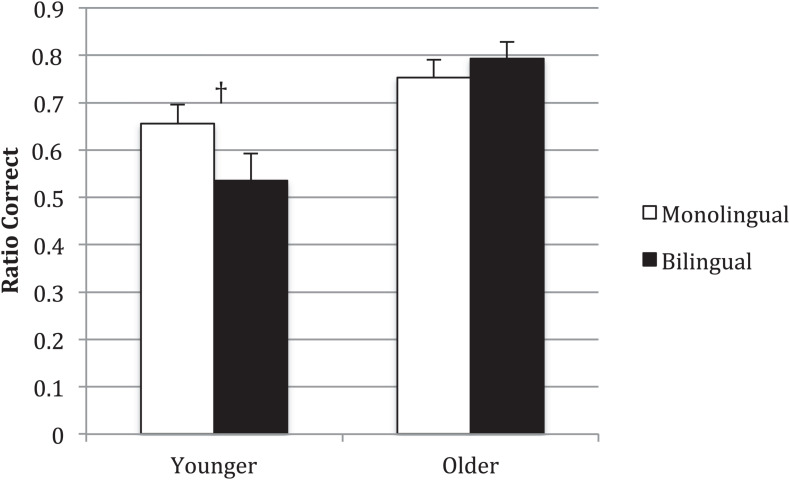
Average ratio correct in English. †*p* = 0.09. Error bars show standard error around the mean.

Figure 1 summarizes the average ratio correct on passives for younger and older monolingual and bilingual children. The younger monolingual children averaged 0.66 (SD = 0.23; 95%CIs [0.58, 0.74]) correct, while the younger bilingual children averaged 0.55 (SD = 0.32; 95%CIs [0.42, 0.63]) correct. The older monolingual children averaged 0.75 (SD = 0.20; 95%CIs [0.68, 0.83]) correct and the older bilingual children 0.79 (SD = 0.19; 95%CIs [0.72, 0.86]). A 2 × 2 (Age Group × Language Group) ANOVA revealed a significant effect for age, *F*(1,120) = 15.92, *p* < 0.001, η^2^_*p*_ = 0.117. The older children’s accuracy was 0.18 higher than the younger children’s (95%CI of this difference [0.09, 0.27]). There was no significant main effect for Language Group, *F*(1,120) = 0.81, *p* = 0.37, η^2^_*p*_ = 0.007 (95%CI of this difference [−0.05, 0.13]).

Usage-based approaches predict an interaction between Language Group and Age Group. In fact, the interaction effect neared significance, *F*(1,120) = 3.25, *p* = 0.07, η^2^_*p*_ = 0.026. To better understand this interaction, we compared the two age groups and the two language groups with independent-samples *t*-tests. In both language groups, the younger children tended to be less accurate than the older children, although this difference approached significance for the monolingual children, *t*(60) = −1.75, *p* = 0.09 (95%CI of this difference [−0.21, 0.01]) and was significant for the bilingual children, *t*(60) = −3.71, *p* < 0.0001 (95%CI of this difference [−0.40, −0.12]). There was a near-significant difference for the younger groups, *t*(64) = 1.72, *p* = 0.09 (95%CI of this difference [−0.02, 0.26]), but not the older groups, *t*(56) = 0.78, *p* = 0.44 (95%CI of this difference [−0.14, 0.06]).

In French, the younger bilingual children scored an average ratio correct of 0.51 (SD = 0.33; 95%CIs [0.40, 0.62]) and the older children 0.68 (SD = 0.31; 95%CIs [0.57, 0.79]). For the younger children, there was no significant difference between languages on a paired *t*-test, *t*(32) = 0.57, *p* = 0.58 (95%CI of this difference [−0.19, 0.11]). In contrast, the older children were significantly more accurate in English than in French, *t*(28) = 2.25, *p* = 0.03 (95%CI of this difference [−0.01, 0.22]).

**FIGURE 2:**
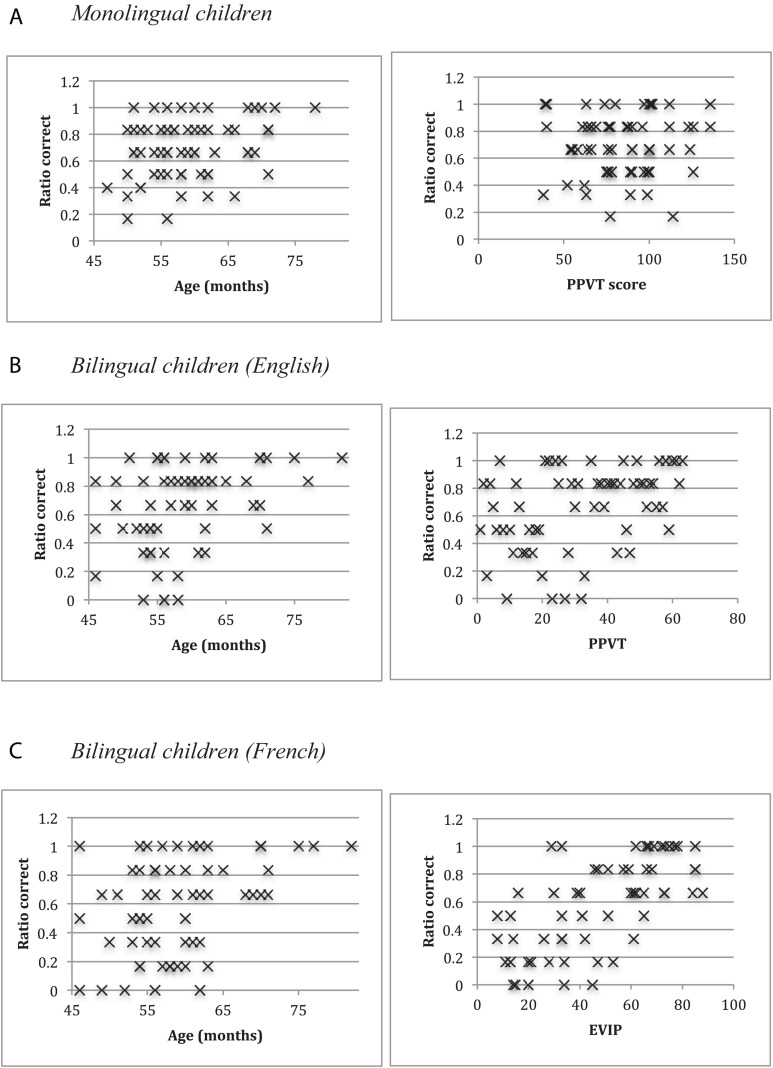
Scatterplots for ratio correct and age **(left)** and vocabulary **(right)**. **(A)** Monolingual children. **(B)** Bilingual children (English). **(C)** Bilingual children (French).

To see how age and vocabulary were related to children’s performance, we correlated age (in months) and raw vocabulary scores with their ratio correct of passives (see Figure 2). For the monolingual children, age showed a trend for being more highly correlated with accuracy, *r*(60) = 0.304, *p* = 0.02, 95%CI [0.059, 0.515], than vocabulary, *r*(60) = 0.043, *p* = 0.74, 95%CI [−0.209, 0.290] (*z* = 1.47, *p* = 0.07). For the bilingual children in English, accuracy was highly correlated with both vocabulary, *r*(55) = 0.500, *p* < 0.0001, 95%CI [0.275-0.673], and age, *r*(60) = 0.377, *p* = 0.003, 95%CI [0.140, 0.573], with no difference between the two correlations (*z* = 0.81, *p* = 0.21). For the bilinguals in French, vocabulary was more highly correlated with accuracy, *r*(58) = 0.685, *p* < 0.00001, 95%CI [0.522, 0.800], than age, *r*(60) = 0.402, *p* = 0.001, 95%CI [0.169, 0.592] (*z* = 2.22, *p* = 0.01). In sum, these correlations revealed that age was an important correlate of accuracy for the monolingual children, while within-language vocabulary scores were an important predictor for the bilingual children.

### Passives: Transposition Errors

**FIGURE 3:**
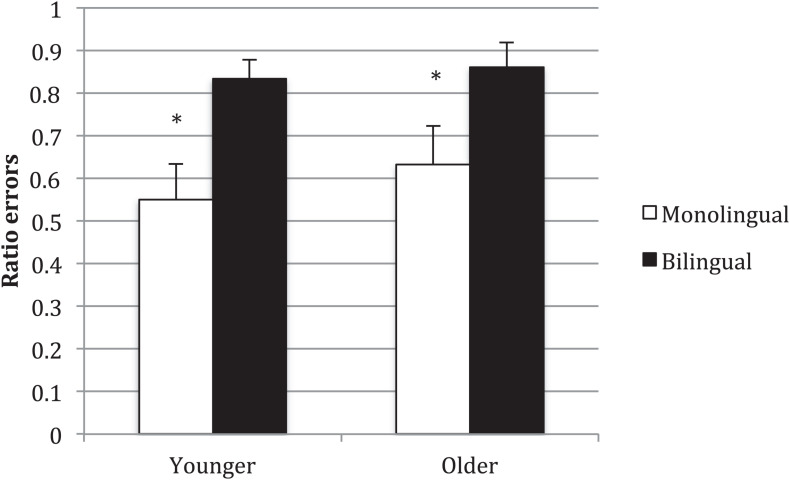
Average ratio transposition errors in English. ^∗^*p* < 0.05. Error bars show standard error around the mean.

We predicted that the monolingual children would make more transposition errors than bilinguals due to their greater vocabulary. Among the monolingual children, 29 of the younger children and 22 of the older children made at least one error. Among the bilingual children, 28 of the younger children and 21 of the older children made at least one error. Figure 3 summarizes the average ratio of errors that were transposition errors (rather than choosing an irrelevant action). The younger monolingual children averaged 0.55 (SD = 0.45; 95%CIs = 0.39, 0.71) transposition errors, while the younger bilingual children averaged 0.83 (SD = 0.24; 95%CIs = 0.75, 0.92). The older monolingual children averaged 0.63 (SD = 0.42; 95%CIs = 0.46, 0.81) transposition errors, while the older bilingual children averaged 0.86 (SD = 0.26; 95%CIs = 0.75, 0.97). A 2 × 2 (Age Group × Language Group) ANOVA showed no effect for Age Group, *F*(1,96) = 0.58, *p* = 0.45, η^2^_*p*_ = 0.006 (95%CI of this difference [−0.09, 0.20]). The main effect for Language Group was significant, *F*(1,96) = 12.56, *p* = 0.001, η^2^_*p*_ = 0.116 (95%CI of this difference [0.11, 0.40]). The interaction effect was not significant, *F*(1,96) = 0.14, *p* = 0.71, η^2^_*p*_ = 0.001. As can be seen in Figure 3, the main effect of Language Group showed that, contrary to predictions, the bilinguals made 0.26 more transposition errors than the monolinguals.

In French, 27 of the younger children and 19 of the older children made at least one error. The younger bilingual children averaged 0.74 transposition errors (SD = 0.28; 95%CIs = 0.62, 0.83) and the older bilingual children 0.87 (SD = 0.25; 95%CIs = 0.76, 0.98). There was no significant difference between languages on paired *t*-tests for either the younger, *t*(26) = 1.14, *p* = 0.25 (95%CI of this difference [−0.08, 0.28]), or the older children, *t*(18) = 1.05, *p* = 0.31 (95%CI of this difference [−0.07, 0.23]).

### Further Tests for Positive Transfer Among the Bilinguals

High positive correlations between languages on accuracy of the interpretation could be evidence for positive transfer. For the younger bilingual children, the correlation between ratio correct in the two languages did not attain significance, *r*(30) = 0.210, *p* = 0.25, 95%CI [−0.150, −0.521]. In contrast, this correlation was positive and significant for the older children *r*(27) = 0.476, *p* = 0.009, 95%CI [0.133, −0.717].

One alternative interpretation to these correlations is that the older bilingual children were simply better at remembering which picture they had chosen in the other language session than the younger bilingual children. To test this possibility, we compared the age groups on the percentage of items for which the children chose the same picture in both languages. The younger children chose the same picture on average 56.6% of the time (SD = 25.7%) and the older children 67.2% (SD = 28.0%). This difference did not reach significance, *t*(60) = 1.57, *p* = 0.12. Thus, it seems unlikely that the older children were simply remembering which picture they had chosen.

## Discussion

Drawing on usage-based approaches, we predicted that younger bilingual children would show no positive transfer in passive constructions in French and English, since they might still be representing only the surface structure of these constructions. In contrast, older children would have an abstract representation of passive constructions and therefore show positive transfer across languages. The results upheld those predictions. We found that the younger bilingual children tended to be less accurate than monolingual children in English and that there was no correlation between languages for the younger bilingual children. In contrast, we found that the 5- to 6-year-old bilingual children tended to be just as accurate in interpreting passive constructions as English monolinguals, even though they had less exposure to English. Furthermore, there were high positive correlations across languages for the older bilingual children.

These results are consistent with usage-based accounts of acquisition proceeding from surface-level to abstract representation. At the age of 3–4 years, the children could be representing passive constructions at the level of words (including the auxiliary and the word “by” or “par”). By the age of 5–6 years, children could be representing at an abstract level, perhaps something like PATIENT-AUXILIARY-PAST PARTICIPLE-“BY/PAR”-AGENT.

According to this interpretation, the way that bilingual children can catch up with monolingual children is through increasing abstraction of syntactic constructions, through exposure to both of their languages. However, our results suggest that there may also be at least two other possible mechanisms (entirely compatible with increasing abstraction) by which bilingual children can catch up with their monolingual counterparts: a semantic bias and selective attention. We consider each of these mechanisms in turn.

One possible mechanism that could allow bilingual children to catch up with monolinguals is that they rely heavily on semantics to interpret language until they acquire the relevant syntactic constructions. In the present study, the finding that even the younger bilinguals were more likely to make transposition errors than the same-aged monolinguals suggests that the bilinguals were taking the meaning of the presented words into account when interpreting the sentences. Some previous research on other linguistic aspects has suggested that young bilingual children can show this sort of semantic bias. For example, one recent study showed that preschool bilingual children interpreted the English past tense morpheme –ed as meaning completion rather than marking for tense (Nicoladis et al., 2020). Future studies can test for this possibility by including irrelevant words in passive sentences to see if bilingual children attempt to find a referent for all the words in their interpretation.

Another possible mechanism (one that is entirely compatible with the semantic bias) is selective attention. The greater rate of transposition errors among bilinguals could mean that the younger monolingual children were not paying as much attention to the words used by the experimenter to select a corresponding picture as the younger bilingual children. Selective attention refers to the ability to pay attention to the relevant aspects of the environment to achieve a goal or solve a problem (Blom et al., 2017). It is possible that even from a young age, bilingual children are selectively attending to the aspects of language that allow them to interpret the meaning. For example, for passives, if they selectively attend to the words contributing to the meaning of the sentence, then, with exposure to more passive sentences in context, they can correct transposition errors quickly. This argument does not necessarily mean that bilinguals would be better than monolinguals at selective attention (although some studies have found this; Blom et al., 2017) but rather that they are relying more on their selective attention in the task of language learning than monolingual children. Analogous results have been reported in other linguistic domains. For example, one study showed that bilingual children relied more on cognitive flexibility when accessing words to tell a story than monolingual children even though they showed no advantage over the monolinguals in cognitive flexibility (Nicoladis and Jiang, 2018). In order to test this interpretation, future studies can include measures of selective attention. If studies show that bilingual children rely more on selective attention in syntactic acquisition than monolinguals, this finding alone would not challenge usage-based approaches. Instead, it would suggest that these approaches need to be supplemented.

One curious finding in the present study was that age was a strong predictor for accuracy for monolingual children, while within-language vocabulary was a strong predictor for accuracy for bilingual children. It is not entirely clear to us why these predictors differ for the two language groups. That within-language vocabulary predicts accuracy fits well with usage-based approaches. That age predicts accuracy for monolingual children suggests that cognitive development may play an important role in monolingual children’s development of passives. If so, it is unclear what aspects of cognitive development would be important and how those aspects of cognitive development would play a role. Again, this finding could be indicative that usage-based approaches may need to be supplemented with the inclusion of some cognitive constructs. In any case, for the moment, we can conclude that vocabulary was not a good measure of the development of passives in monolingual children in this study.

Our characterization of children’s abstract representation (i.e., PATIENT-AUXILIARY-PAST PARTICIPLE-“BY/PAR”-AGENT) is highly speculative; the exact nature of abstract representation in usage-based approaches is rarely spelled out (Tomasello, 2000; Goldberg, 2013). In the present study, we included constructions that are word-for-word translations of each other, meaning that the word order is exactly the same in French and English. It is not clear that this perfect transliteration is necessary for bilinguals to show positive transfer. In fact, the weight of evidence to date suggests that adult bilinguals can show positive transfer even when word order varies across languages (Hatzidaki et al., 2011; Hwang et al., 2018). One case study of a bilingual child also suggested that positive transfer could occur, despite a lack of similarities in the constructions in the two languages (Babatsouli and Nicoladis, 2019). If positive transfer can occur even in the absence of similar word orders, then the form of abstract representation might be primarily in terms of function. Tomasello (2000) argued that the abstract representation would critically be based on constructions that serve highly similar communicative functions. Future research could therefore focus on how passives are used in communication, as well as focusing on linguistic constructions that differ across languages.

There were a number of limitations to the present study. First, only six passives sentences were included. Second, to test for the effects of age, we did a median split with children aged 3–6 years, rather than recruit participants with a greater difference in age groups. Both of these choices may have reduced the statistical power, and we may therefore be underestimating the true difference. Another limitation is that, in French, we used a translated version of the passive sentences. The bilingual children in the present study tended to do worse on the French version than the English version, particularly the older children. As we had no French monolingual comparison group, we do not know if this tendency is due to the bilingual children’s poorer French (than English) performance or whether there were some weaknesses to our translated version. Future research can be designed with greater statistical power and include comparison groups of monolinguals in both languages.

In conclusion, we have shown here that there are developmental changes in bilingual children’s positive transfer across languages in passive constructions. These results are consistent with the argument that children’s representation of linguistic constructions becomes increasingly abstract as they learn to use their language(s). We have also found suggestive evidence that bilinguals may better employ selective attention to the task of learning passives than monolinguals. With increasingly abstract representation and skillful employment of selective attention, the bilingual children in this study performed better in a language than expected from their exposure time.

## Data Availability Statement

The original contributions presented in the study are included in the article/Supplementary Material, further inquiries can be directed to the corresponding author.

## Ethics Statement

The studies involving human participants were reviewed and approved by the University of Alberta Research Ethics Board. Written informed consent to participate in this study was provided by the participants’ legal guardian/next of kin.

## Author Contributions

EN spearheaded the data collection, analyzed the data, and wrote the manuscript. SS reviewed the literature, coded the data, and edited the manuscript. Both authors contributed to the article and approved the submitted version.

## Conflict of Interest

The authors declare that the research was conducted in the absence of any commercial or financial relationships that could be construed as a potential conflict of interest.

## Figures and Tables

**TABLE A1:** Passive sentences used in English and French.

**English**	**French**
The boy is chased by the dog.	*Le garçon est chassé par le chien.*
The mother is fed by the baby.	*La maman est nourrie par le bébé.*
The cat is bitten by the dog.	*Le chat est mordu par le chien.*
The girl is hugged by the monkey.	*La fille est câlinée par le singe.*
The elephant is carried by the boy.	*L’éléphant est porté par le garçon.*
The baby is pushed by the mother.	*Le bébé est poussé par la maman.*
